# Effect of Household Type on the Prevalence of Metabolic Syndrome in Korea: Using Propensity Score Matching

**DOI:** 10.3390/healthcare10101894

**Published:** 2022-09-28

**Authors:** Jisu Park, Ilsu Park

**Affiliations:** 1 Busan Eastern Branch of Korea Association of Health Promotion, 145 Chungnyeoldaero, Dongnae-gu, Busan 47734, Korea; 2 Department of Healthcare Management, Dong-eui University, 176 Eomgwangro, Busanjin-gu, Busan 47340, Korea

**Keywords:** KNHANES, household types, metabolic syndrome, propensity score matching

## Abstract

This study analyzed the effect of the household type on the prevalence of metabolic syndrome in Koreans utilizing data from the sixth, seventh, and eighth Korea National Health and Nutrition Examination Surveys conducted by the Korea Disease Control and Prevention Agency from 2015 to 2019. The demographic characteristics, metabolic syndrome characteristics according to household type, and risk factors of 25,092 subjects were identified using the Rao–Scott *χ*^2^–test and weighted multiple logistic regression results. Furthermore, to understand the effect of the household type on prevalence of metabolic syndrome, the selection bias between the groups was eliminated using the propensity score matching method. The average treatment effect for those treated for metabolic syndrome prior to propensity score matching was higher for single-person households, with 0.353 and 0.268 for single- and multi-person households, respectively. The difference was statistically significant (*p* < 0.0001). However, after propensity score matching, it was observed to be higher for multi-person households, with 0.290 and 0.316 for single- and multi-person households, respectively. However, the difference was not statistically significant (*p* < 0.1822). Difference was observed regarding the prevalence of metabolic syndrome by individual characteristics, some of which were considered in previous studies. However, the household type alone did not explain the prevalence of metabolic syndrome.

## 1. Introduction

According to the Statistics Korea census, single-person households refer to homes in which one person independently maintains a livelihood, such as cooking and sleeping. These accounted for 9.0% of all households in 1990 among the peripheral household type. However, this proportion sharply increased to 15.5% in 2000, 27.2% in 2015, and 31.7% in 2020. Currently, single-person households have become commonplace and constitute the second highest proportion of household types, following two-generation households [1]. This trend is expected to continue in the future. According to the prospective household projection by Statistics Korea, the proportion of single-person households in Korea is predicted to reach 32.3% and 37.1% by 2025 and 2045, respectively [2]. This increase is a global trend. As of 2013, the proportions of single-person households were 32.4% and 22.0% in Japan and Taiwan, respectively. Furthermore, over one-third of the total households in European countries, such as Norway, Finland, Denmark, Switzerland, and Germany, were also single-person households [3]. Government policies and housing and food markets are already changing and developing to accommodate these households in Europe and the United States, where their numbers have been increasing substantially [4,5]. Similarly, Korea is preparing strategies to respond to the health risks posed by single-person households by establishing an integrated support system at the national level and promoting mental health improvement by providing opportunities for social participation [6]. According to previous studies, changes in the household type significantly influence individual health behavior [7]. The level of physical and mental health was lower in single-person households than in multi-person households [8]. The chronic disease rate, experience rate of ambulatory care, and admission rate were also higher for the former than the latter; similarly, suspected rates of depression and suicidal ideation are three and four times higher, respectively, in single-person households than in multi-person ones [6]. Furthermore, prior studies have demonstrated that eating alone increases the likelihood of nutritional imbalance and obesity [9]. Metabolic syndrome, the target disease of the present study, refers to a frequent occurrence of abdominal obesity, dyslipidemia, hypertension, and hyperglycemia. The rate of cardiovascular diseases, the main cause of death in Korean adults, is higher among Korean adults with metabolic syndrome. For this reason, many countries, including Korea, currently consider metabolic syndrome as a major target disease in chronic disease management projects [10,11]. In domestic research that compares metabolic syndrome’s risk by household type, single-person households aged over 30 years had a risk of metabolic syndrome that was 1.78-times higher than multi-person households [12]. Other domestic studies have demonstrated that demographic characteristics, such as gender, age [13], marital status, basic livelihood security, and socioeconomic level [14], are related to the prevalence of metabolic syndrome [15,16,17,18,19]. Health behaviors, such as smoking and drinking [20], diet and exercise [21], physical activity, skipping breakfast, eating out, dietary supplement intake, use of nutrition labels, stress, and sleep [22], have also been found to be associated with the prevalence of metabolic syndrome [15,18,19,20,23,24].

As described previously, many studies have been conducted on the health status and behaviors of single-person households and their effects on the prevalence of diseases, such as metabolic syndrome. Nevertheless, since these studies examined the relationship between the prevalence of metabolic syndrome and household type with various factors, in-depth research on how the household type affects the prevalence of metabolic syndrome remains limited. Thus, this study examined the difference in the prevalence of metabolic syndrome according to household type by applying propensity score matching (PSM) to verify the impact of household type on metabolic syndrome more precisely. Furthermore, the study intended to provide the basic data necessary to suggest the direction of health management policies and systems related to the management of metabolic syndrome.

## 2. Materials and Methods

### 2.1. Subjects and Data Collection

This study employed raw public data from the sixth, seventh, and eighth Korea National Health and Nutrition Examination Surveys (KNHANES) conducted by the Ministry of Health and Welfare and Korea Disease Control and Prevention Agency from 2015 to 2019. The data were integrated according to the data integration method between the periods based on the guidelines for analyzing the KNHANES raw data. Moreover, the total weighted *n* was calculated and used for the analysis.

Based on the inter-period data integration, there were 39,759 subjects. Of these, 25,092 were selected as the final study subjects, excluding 11,741 aged below 30 years based on the survey questions’ characteristics and the study’s nature, and 2926 with outliers and missing values.

### 2.2. Research Variables

Since the research aimed to determine the effect of household type on the prevalence of metabolic syndrome, the dependent variable was set as the presence or absence of metabolic syndrome. According to the guidelines of the modified National Cholesterol Education Program Adult Treatment Panel III of the American Heart Association and the National Heart, Lung, and Blood Institute, metabolic syndrome is defined as a case in which three or more of the following five conditions are present: abdominal obesity (defined as ≥90 cm for males and ≥85 cm for females, as per the 2005 standards presented by the Korean Society for the Study of Obesity), hypertriglyceridemia, low HDL cholesterol, hypertension, and hyperglycemia. Hypertriglyceridemia refers to a blood triglyceride level of 150 mg/dl or above. Furthermore, low HDL cholesterol is delineated as 40 mg/dl or lower and 50 mg/dl or lower in males and females, respectively. Hypertension was defined as a systolic blood pressure of 130 mmHg or higher, a diastolic blood pressure of 85 mmHg or higher, or taking antihypertensive medications. Hyperglycemia was defined as a fasting blood sugar level of 100 mg/dl or above, being diagnosed with diabetes by a physician, taking diabetes medications (insulin shots), or consuming hypoglycemic agents.

Furthermore, factors affecting the prevalence of metabolic syndrome were composed of the household type; demographic characteristics including gender, age, marital status, and eligibility for the National Basic Living Security Program; and health behaviors included smoking, drinking, aerobic physical activity, skipping breakfast, eating out, tendency to use nutrition labels when buying or choosing processed foods, and stress in daily life (as an indicator of mental health state). These factors were used to identify the characteristics of the subjects and used as the matching variables for the PSM to eliminate heterogeneity between the comparison groups except for household type. The detailed description is shown in Table 1.

### 2.3. Statistical Analysis

As mentioned previously, this study aimed to understand the pure influence of household type on the prevalence of metabolic syndrome. Generally, randomized controlled trials are the most effective research method to confirm the causal effect between the influencing factors and response results [25]. However, it is practically impossible to perform research that controls the group in advance (i.e., an experimental study) in a comparative investigation between groups using secondary data. When conducting research using non-experimental or observational data, most studies fail to overcome the issue of selection bias and endogeneity in the selection of groups compared as the differences between the comparison groups are disregarded [26]. Consequently, most existing studies had to either stratify factors to use them for analysis or estimate the explanatory variables’ effects through statistical corrections, such as multiple regression analysis. However, the existing stratification analysis essentially does not work if many covariates are considered; furthermore, problems, such as multicollinearity, may arise if all variables are included in the covariate in the multiple regression analysis [27]. PSM, a statistical method proposed to overcome this limitation, is a technique proposed by Rosenbaum and Rubin to statistically create a “control group” similar to an “experimental” one [25]. Ultimately, the PSM is a counterfactual model that can reduce bias in estimating the treatment effect of non-experimental data [28]. It is also known as the potential outcome model.

The first step in PSM is to pair individuals with similar propensity scores to decrease the imbalance of covariates between the experimental and control groups. The most commonly used stratified matching technique is nearest neighbor 1: *n* matching, which pairs subjects that have the same or similar propensity scores. Moreover, caliper matching uses a value corresponding to one-fourth of the standard error of the estimated propensity score for matching. Radius matching corresponds to the control group within a preset interval from the experimental group’s propensity score. Kernel matching determines the weight by a value inversely proportional to the difference in the propensity scores between the experimental and control groups and pairs the former’s subjects based on the latter’s weighted average. Mahalanobis metric matching pairs with the smallest Mahalanobis distance between the experimental and control groups. Optimal matching matches subjects based on network flow theory and has been widely used in recent years [29]. After the second PSM, a *t*-test is performed before and after matching to understand its effect. Furthermore, the imbalance of the covariates could be checked by obtaining the standardized mean difference (SMD); this value ranges from –100% to 100%. The closer the value to 0%, the smaller the imbalance; additionally, it is generally recommended to be less than 10% [30]. The average treatment effect for the treated (ATT) is calculated for groups that are finally matched; ATT refers to the counterfactual difference between the results when the subject is and is not exposed to a factor.

In this study, the effects of the influencing factors, such as household type, four demographic characteristics, nine health behavior characteristics, and stress for mental health, on the prevalence of metabolic syndrome were examined through a weighted multiple regression. This was performed to comprehensively review the factors affecting the prevalence of metabolic syndrome reviewed in the previous studies and compare the pure effect of household type on the prevalence of metabolic syndrome.

**Figure 1:**
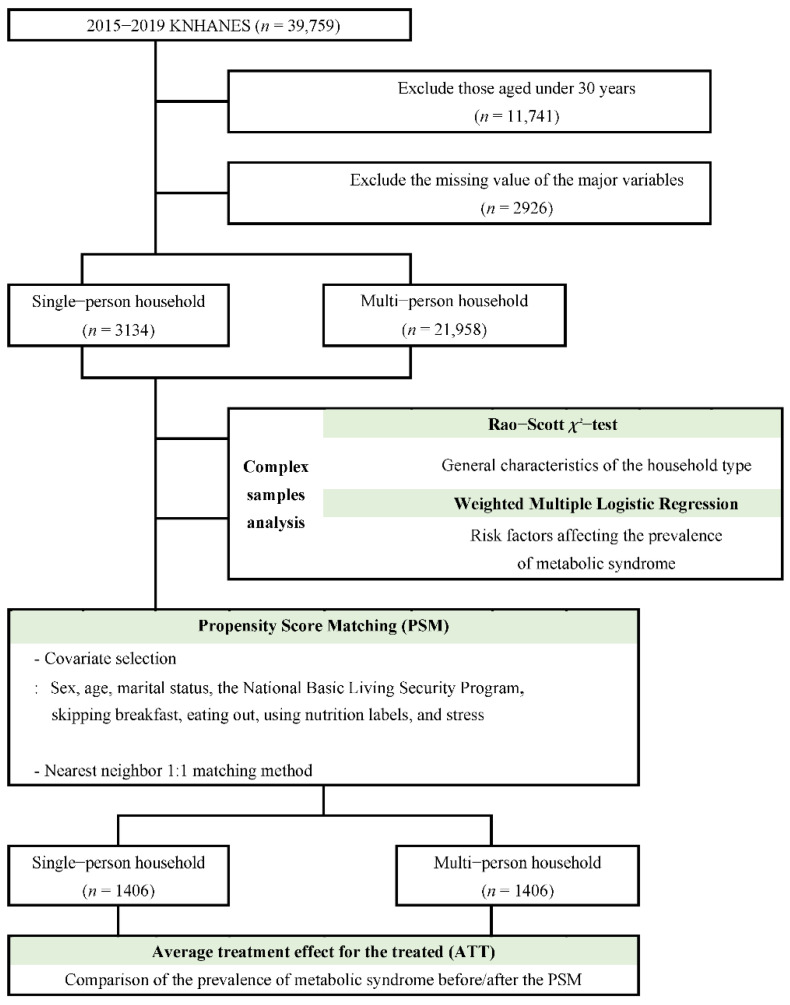
The conceptual framework of the research.

Among the aforementioned factors, all except household type were set as the matching variables. Nearest neighbor 1:1 matching was employed as the matching method; moreover, gender, considered to have the greatest influence on disease prevalence, was matched first, followed by the remaining factors. Subsequently, the SMD before and after matching was compared to verify the balance between the two matched groups, and the statistical difference between them was assessed using the Rao–Scott *χ*^2^-test. Each factor presented a percentage bias reduction, which is the reduction percentage of a selection bias, to suggest the amount of heterogeneity reduction between the comparison groups. Furthermore, after removing the selection bias between the two comparison groups, the ATT value was presented to confirm the prevalence of metabolic syndrome purely based on household type (Figure 1).

The Rao−Scott *χ*^2^− test, weighted multiple regression, and the ATT analysis used in this study were performed as a complex sample analysis; SAS 9.4 (SAS, Inc., Cary, NC, USA) was used for the analysis.

## 3. Results

### 3.1. General Characteristics of the Subjects

The study included 25,092 (weighted *n*: 32,334,413) final subjects, comprising 3134 (weighted *n*: 3,251,512) and 21,958 (weighted *n*: 29,082,901) single- and multi-person household subjects, respectively. Table 2 shows the distribution by demographic and social characteristics, health behavior characteristics, mental health, and metabolic syndrome.

The differences in the distribution of the subjects’ general characteristics by household type were statistically analyzed using the Rao–Scott *χ*^2^–test. Consequently, the distribution of the single- and multi-person households according to all demographic characteristics demonstrated a statistically significant difference. Among the health behavior characteristics, there was no statistically significant difference in the use of nutrition labels when buying or choosing processed foods according to household type (*p* = 0.1277). There was also no statistically significant difference in stress, an indicator of mental health, according to household type (*p* = 0.5629). Nonetheless, the prevalence of metabolic syndrome, the main interest of this study, was 35.3% and 26.8% for single- and multi-person households, respectively, signifying that the prevalence was higher in the former by 8.5%; this difference was statistically significant (*p* < 0.0001).

### 3.2. Analysis of the Factors Affecting the Prevalence of the Metabolic Syndrome

Logistic regression was performed according to the complex sample analysis method to analyze the effects of factors reviewed in previous studies, including demographic characteristics, health behavior features, and mental health factors, in addition to the household type, on the prevalence of metabolic syndrome (Table 3).

Among the factors used for the analysis, gender, marital status, smoking, skipping breakfast, and stress were found to have a significant impact on the prevalence of metabolic syndrome. Specifically, the risk of metabolic syndrome was higher in men than in women (OR = 0.56). Furthermore, as age increased by one year, the risk increased by 1.51 times. The risk of metabolic syndrome was also higher by 1.34, 1.21, 1.19, and 1.29 times for those subjects receiving the National Basic Living Security Program, indirectly suggesting their economic status, who did not exercise, who skipped breakfast, and who were undergoing mental stress, respectively. The risk of metabolic syndrome was 1.02-times greater in single-person than in multi-person households; however, this difference was not statistically significant (OR = 1.02, 95% CI 0.87–1.19).

### 3.3. Propensity Score Matching

**Figure 2:**
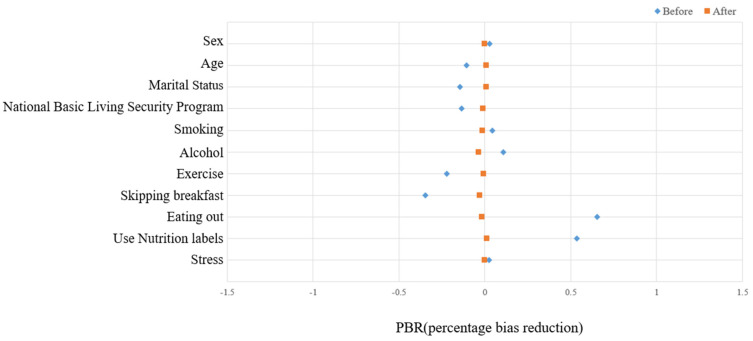
Covariates imbalance before and after propensity score matching.

Figure 2 and Table 4 show the results of PSM conducted to remove heterogeneity between the two groups, namely, the experimental “single-person household” and the control “multi-person household” groups. The groups were compared to understand the effect of household type on the prevalence of metabolic syndrome.

A close review of the PSM results reveals that heterogeneity is exceedingly high before matching all factors (*p* < 0.0001), except for the use of nutrition labels and stress factors. However, it could be confirmed that heterogeneity due to the household type was removed from all matching factors after matching (*p* > 0.05), except for the age factor (*p* < 0.0001). Specifically, the SMD decreased after matching all factors, and the PBR was approximately 65.0% or more.

The number of subjects analyzed through the final PSM included 1406 single- (weighted *n*: 1,667,732) and multi-person (weighted *n*: 1,817,642) household subjects each.

Figure 2 presents the covariate imbalance before and after propensity score matching.

### 3.4. Analysis of the Difference in the Prevalence of Metabolic Syndrome by Household Type before and after PSM

The ATT refers to the counterfactual difference between the outcome of the subjects exposed to a factor (in the case of “single-person households”) and that of the “identical” individuals unexposed to it (in the case of “multi-person households”). In this study, the pure effect of household type on metabolic syndrome prevalence was analyzed in terms of the ATT after removing heterogeneity between the comparison groups for the factors influencing the metabolic syndrome prevalence in addition to household type (Table 5).

Consequently, the ATT for metabolic syndrome before the PSM was higher in single-person households at 0.353 compared with multi-person households at 0.268; this difference was statistically significant (difference = 0.086, *p* < 0.0001). However, the ATT for metabolic syndrome after the PSM was indicated to be greater in the multi-person households at 0.316 compared with single-person households at 0.29; nevertheless, this was not statistically significant (difference = −0.026, *p* = 0.1822). Specifically, since this was the analysis result after removing the factors that affect the prevalence of metabolic syndrome other than the household type for the two groups and securing homogeneity between the two groups more clearly, it was suggested that the household type alone had no statistically significant effect on the prevalence of metabolic syndrome.

## 4. Discussion

The paradigm shift in diseases from acute diseases in the past to chronic diseases, such as hypertension, diabetes, and metabolic syndrome, which have the combined characteristics of both, has caused many countries to focus on chronic disease management policies to facilitate positive modifications in citizens’ health behaviors and lifestyles. Furthermore, the most common household type has shifted from a multi-person to a single-person household due to population aging and the decline in birth rates. Studies have reported that this change is closely associated with the prevalence of chronic diseases [7]. However, most existing studies have been unable to compare the chronic-disease-related characteristics and prevalence according to household type because the comparison groups’ characteristics were mixed. Thus, it was difficult to clearly understand the pure effect of household type on the prevalence of chronic diseases. Considering this challenge, the present study used the data from the sixth, seventh, and eighth KNHANES conducted by the Korea Disease Control and Prevention Agency from 2015 to 2019 to investigate the effect of the sociodemographic characteristics, health behavior characteristics, mental health factors, and household type on the prevalence of metabolic syndrome in 25,092 (3134 and 21,958 single- and multi-person household subjects, respectively) adults aged over 30 years. To overcome the limitations, such as bias and endogeneity between the comparison groups that were not considered in previous studies, the factors that could affect the prevalence of metabolic syndrome other than household type were controlled using PSM to determine the pure effect of the household type on the prevalence of chronic diseases.

Furthermore, before performing the aforementioned analysis, a weighted multiple logistic regression utilized in the analysis of the relationship between the risk factors and metabolic syndrome prevalence was conducted. This regression analysis compared the results to those of the previous research that identified the relationship between the prevalence of metabolic syndrome and various health risk characteristics and to this study’s final outcome, which identified the effect of household type alone on the prevalence of metabolic syndrome. Accordingly, gender, age, eligibility for the National Basic Living Security Program, which is a proxy variable for the subjects’ economic status, smoking, exercise, skipping breakfast, and stress had a statistically significant effect on the prevalence of metabolic syndrome. Although some differences in the level of influence on metabolic syndrome prevalence depending on the subject and method of measuring variables may were found in this study, the results were generally consistent with previous studies which reported that individual characteristics could influence the prevalence of metabolic syndrome [31,32,33,34,35,36,37,38,39].

However, regarding the household type, the risk of metabolic syndrome was 1.02 times higher in single-person than in multi-person households; moreover, the difference was not statistically significant. This was consistent with the ATT analysis results, which showed that the household type alone did not have a statistically significant effect on the prevalence of metabolic syndrome. These results are also consistent with those of the weighted multiple regression considering all the factors affecting metabolic syndrome analyzed previously. Nonetheless, the reason differences were observed in the prevalence of metabolic syndrome by household type demonstrated in some previous studies and in this study’s general characteristics analysis was thought to be because of the effect of differences in health behaviors and eating habits of family members that may change depending on the household type.

Although the study aimed to identify and compare the factors affecting the prevalence of metabolic syndrome and to provide basic data for its prevention and management, the following limitations exist. First, this was a secondary study using data collected at the national level for various purposes, which may be inappropriate for employment as measured variables that meet the research objective. Nonetheless, because of the study’s use of large-scale survey data conducted at the national level, the reliability and accuracy of the findings could be considered exceedingly high. Second, the results depending on the actual change in household type could not be confirmed as this study was cross-sectional, utilizing the phenomenon only at a specific time. If the relationship between the changes in the household type and the prevalence of metabolic syndrome could be identified through a future longitudinal study, its aim could be presented more clearly.

## 5. Conclusions

A difference was observed in the prevalence of metabolic syndrome by individual characteristics, some of which were considered in previous studies; however, the household type alone did not affect the prevalence of metabolic syndrome. This is because individual characteristics that may vary depending on household type had a greater impact on the prevalence of metabolic syndrome than the household type alone. Although this study is similar to previous studies in terms of results, it is meaningful because the findings were derived through a statistical methodology that ensured strict control between comparison groups. Furthermore, based on the outcomes of this study, establishing an individual health-promotion strategy for drinking, smoking, aerobic physical activity, and stress control is expected to be more effective for the prevention of metabolic syndrome than policies and education for healthy living based on household type.

## Figures and Tables

**Table 1:** Definition of the study variables.

Factor	Variable	Description
Dependent variable	Metabolic syndrome	Three or more diagnostic criteria for metabolic syndrome
Independent variable	Household type	Single-person household, multi-person household (two or more family members)
Demographic characteristics	Gender	Male, female
	Age	Over 30 years old
	Marital status	Married, unmarried
	National Basic Living Security Program	Yes (previous or current), No
Health behavior characteristics	Smoking	Smoked 5 packs (100 cigarettes) or more in his/her life and currently smoking
	Drinking	History of drinking more than once a month in the past year
	Aerobic physical activity	Moderate-intensity physical activity for at least 2 h and 30 min, high-intensity physical activity for at least 1 h and 15 min, or a mix of moderate- and high-intensity physical activities for a proportionate amount of time during the week
	Skipping breakfast	History of eating breakfast in the past year
	Eating out	Eating out more than once a week
	Using nutrition labels	Using nutrition labels when buying or choosing processed foods
Mental health	Stress	Stress in daily life

**Table 2:** General characteristics among the single- and multi-person households.

Factors			Single-Person Household				Multi-Person Household				Rao–Scott *χ*^²^	*p*- Value
			*n*	(%)	*N* ^a^	(%)	*n*	(%)	*N* ^a^	(%)		
Total			3134	(100.0)	3,251,512	(100.0)	21,958	(100.0)	29,052,901	(100.0)		
Demographic and social characteristics	Sex	Male	1131	(36.1)	1,469,241	(45.2)	9656	(44.0)	14,220,000	(49.0)	8.17	<0.0001
		Female	2003	(63.9)	1,782,271	(54.8)	12,302	(56.0)	14,830,000	(51.1)		
	Age (Mean ± SD)		63.5 ± 0.4		58.8 ± 0.5		54.1 ± 0.2		51.4 ± 0.2		949.29	<0.0001
	Marital Status	Married	2529	(80.7)	2,299,644	(70.7)	20,889	(95.1)	27,220,000	(93.7)	822.08	<0.0001
		Unmarried	605	(19.3)	951,868	(29.3)	1069	(4.9)	1,837,243	(6.3)		
	National Basic Living Security Program	Yes	613	(19.6)	559,566	(17.2)	1112	(5.1)	1,395,204	(4.8)	411.07	<0.0001
		No	2519	(80.4)	2,691,069	(82.8)	20,843	(94.9)	27,650,000	(95.2)		
Health behavior characteristics	Smoking	Yes	618	(19.9)	846,511	(26.2)	3616	(16.5)	5,660,882	(19.6)	37.66	<0.0001
		No	2488	(80.1)	2,381,306	(73.8)	18,251	(83.5)	23,280,000	(80.4)		
	Alcohol	Yes	1320	(42.4)	1,621,795	(50.2)	11,658	(53.3)	16,510,000	(57.0)	31.88	<0.0001
		No	1790	(57.6)	1,609,111	(49.8)	10,217	(46.7)	12,440,000	(43.0)		
	Exercise	Yes	1060	(34.1)	1,222,139	(37.8)	9202	(42.0)	12,740,000	(44.0)	26.83	<0.0001
		No	2051	(65.9)	2,010,005	(62.2)	12,698	(58.0)	16,240,000	(56.0)		
	Skipping breakfast	No	2481	(87.6)	2,435,469	(83.9)	17,259	(89.4)	21,910,000	(87.6)	17.44	<0.0001
		Yes	351	(12.4)	465,968	(16.1)	2037	(10.6)	3,095,662	(12.4)		
	Eating out	Yes	1651	(58.3)	1,893,088	(65.3)	13,728	(71.2)	18,950,000	(75.8)	93.76	<0.0001
		No	1181	(41.7)	1,008,349	(34.8)	5567	(28.9)	6,053,438	(24.2)		
	Using nutrition labels	Yes	432	(29.8)	554,708	(32.3)	4922	(34.8)	6,738,336	(34.9)	2.32	0.1277
		No	1018	(70.2)	1,164,419	(67.7)	9232	(65.2)	12,560,000	(65.1)		
Mental health	Stress	Yes	758	(24.4)	846,513	(26.2)	5569	(25.5)	7,763,267	(26.8)	0.33	0.5629
		No	2347	(75.6)	2,058,370	(63.3)	16,293	(74.5)	21,170,000	(73.2)		
Disease	Metabolic syndrome	Yes	1192	(38.0)	1,148,837	(35.3)	6243	(28.4)	7,772,633	(26.8)	65.94	<0.0001
		No	1942	(62.0)	2,102,675	(64.7)	15,715	(69.7)	21,280,000	(73.3)		

Note: ^a^ Weighted *n.*

**Table 3:** Impact of the factor prevalence of metabolic syndrome using a complex survey regression.

Factors			MS	
			OR ^a^	95% CI ^b^
Demographic and social characteristics	Sex	Male (Ref)	1	-
		Female	0.56 ***	(0.50–0.62)
	Age		1.51 ***	(1.45–1.57)
	Marital status	Married (Ref)	1	-
		Unmarried	1.09	(0.89–1.33)
	National Basic Living Security Program	Yes	1.34 **	(1.09–1.65)
		No (Ref)	1	-
Health behavior characteristics	Smoking	Yes	1.24 **	(1.10–1.41)
		No (Ref)	1	-
	Alcohol	Yes	1.04	(0.94–1.15)
		No (Ref)	1	-
	Exercise	Yes	1	-
		No	1.21 **	(1.10–1.33)
	Skipping breakfast	No (Ref)	1	-
		Yes	1.19 *	(1.03–1.36)
	Eating out	Yes	0.90	(0.80–1.01)
		No (Ref)	1	-
	Using nutrition labels	Yes (Ref)	1	-
		No	1.07	(0.97–1.18)
Mental health	Stress	Yes	1.29 ***	(1.16–1.42)
		No (Ref)	1	-
	Household type	One person	1.02	(0.87–1.19)
		Multi person (Ref)	1	-

Note: ^a^ OR = Odds Ratio; ^b^ 95% Confidence Interval; * *p* < 0.05; ** *p* < 0.01; *** *p* < 0.001.

**Table 4:** Comparison between the unadjusted and adjusted means of covariates on the metabolic syndrome prevalence.

Factors			Before Matching (*n* = 25,092)				SMD ^b^	*p-* Value	After Matching (*n* = 2812)				SMD	*p-* Value	PBR ^c^
			Single-Person Household		Multi-Person Household				Single-Person Household		Multi-Person Household				
			*N* ^a^	(%)	*N* ^a^	(%)			*N* ^a^	(%)	*N* ^a^	(%)			
Total			3,251,512	(100.0)	29,052,901	(100.0)			1,667,732	(100.0)	1,817,642	(100.0)			
Demographic and social characteristics	Sex	Male	1,469,241	(45.2)	14,220,000	(49.0)	0.02	<0.0001	832,963	(50.0)	832,787	(45.8)	0.00	0.0757	100.0
		Female	1,782,271	(54.8)	14,830,000	(51.1)			834,768	(50.1)	984,855	(54.2)			
	Age (Mean ± SD)		58.8 ± 0.5		51.4 ± 0.2		0.54	<0.0001	52.8 ± 0.6		53.6 ± 0.5		0.01	<0.0001	97.7
	Marital status	Married	2,299,644	(70.7)	27,220,000	(93.7)	0.65	<0.0001	1,028,450	(61.7)	1,181,028	(65.0)	−0.02	0.1735	97.6
		Unmarried	951,868	(29.3)	1,837,243	(6.3)			1,667,732	(38.3)	636,613	(35.0)			
	National Basic Living Security Program	Yes	559,566	(17.2)	1,395,204	(4.8)	−0.35	<0.0001	174,624	(10.5)	183,081	(10.1)	−0.03	0.7871	91.1
		No	2,691,069	(82.8)	27,650,000	(95.2)			1,493,108	(89.5)	1,634,560	(89.9)			
Health behavior characteristics	Smoking	Yes	846,511	(26.2)	5,660,882	(19.6)	−0.22	<0.0001	491,132	(29.5)	514,194	(28.3)	−0.01	0.6018	95.9
		No	2,381,306	(73.8)	23,280,000	(80.4)			1,176,599	(70.6)	1,303,447	(71.7)			
	Alcohol	Yes	1,621,795	(50.2)	16,510,000	(57.0)	0.11	<0.0001	949,294	(56.9)	859,903	(47.3)	−0.04	0.0669	67.1
		No	1,609,111	(49.8)	12,440,000	(43.0)			718,437	(43.1)	957,739	(52.7)			
	Exercise	Yes	1,222,139	(37.8)	12,740,000	(44.0)	0.04	<0.0001	755,371	(45.3)	804,876	(44.3)	−0.02	0.6516	64.6
		No	2,010,005	(62.2)	16,240,000	(56.0)			912,360	(54.7)	1,012,766	(55.7)			
	Skipping breakfast	No	2,435,469	(83.9)	21,910,000	(87.6)	−0.14	<0.0001	1,341,499	(80.4)	1,459,448	(80.3)	−0.01	0.9384	92.6
		Yes	465,968	(16.1)	3,095,662	(12.4)			326,232	(19.6)	358,193	(19.7)			
	Eating out	Yes	1,893,088	(65.3)	18,950,000	(75.8)	−0.15	<0.0001	1,308,435	(78.5)	1,395,513	(76.8)	0.01	0.3419	95.5
		No	1,008,349	(34.8)	6,053,438	(24.2)			359,297	(21.5)	422,129	(23.2)			
	Using nutrition labels	Yes	554,708	(32.3)	6,738,336	(34.9)	−0.11	0.1277	544,393	(32.6)	553,067	(30.4)	0.01	0.3241	91.4
		No	1,164,419	(67.7)	12,560,000	(65.1)			1,123,338	(67.4)	1,264,574	(69.6)			
Mental health	Stress	Yes	846,513	(26.2)	7,763,267	(26.8)	0.03	0.5629	441,562	(26.5)	477,272	(26.3)	0.00	0.9037	94.2
		No	2,380,480	(73.8)	21,170,000	(73.2)			1,226,169	(73.5)	1,340,369	(73.7)			

Note: ^a^ Weighted *n*; ^b^ SMD (Standardized Mean Difference); ^c^ PBR (Percentage Bias Reduction).

**Table 5:** Average treatment effect on the treatment of metabolic syndrome using PSM.

Diseases	Matching	Mean		Difference (A-B)	95% CI ^a^ of Difference		*t*- Statistics	*p*-Value
		Single-Person Household (A)	Multi-Person Household (B)		Lower Limit	Upper Limit		
MS	Before	0.353	0.268	0.086	0.0635	0.1081	7.56	<0.0001
	After	0.290	0.316	−0.026	−0.0648	0.0123	−1.34	0.1822

Note: ^a^ 95% Confidence Interval.

## Data Availability

Data are available on the official KNHANES website: https://knhanes.kdca.go.kr/knhanes/sub03/sub03_02_05.do (accessed on 1 July 2022).
